# Subgingival microbiota of dogs with healthy gingiva or early periodontal disease from different geographical locations

**DOI:** 10.1186/s12917-020-02660-5

**Published:** 2021-01-06

**Authors:** C. Wallis, L. Milella, A. Colyer, C. O’Flynn, S. Harris, L. J. Holcombe

**Affiliations:** 1 WALTHAM Petcare Science Institute, Mars Petcare UK, Melton Mowbray, Leicestershire UK; 2 The Veterinary Dental Surgery, Byfleet, Surrey, UK

**Keywords:** Microbiota, Plaque, Periodontal disease, Gingivitis, Canine, Geography

## Abstract

**Background:**

Periodontal disease is the most common oral disease of dogs worldwide and results from a complex interplay between plaque bacteria, the host and environmental factors. Recent studies have enhanced our understanding of the associations between the plaque microbiota and canine periodontal disease. These studies, however, were limited in their geographical reach. Thus associations between the canine oral microbiota and geographical location were investigated by determining the composition of subgingival plaque samples from 587 dogs residing in the United Kingdom (UK), United States of America (USA), China and Thailand using 454-pyrosequencing.

**Results:**

After quality filtering 6,944,757 sequence reads were obtained and clustering of these at ≥98% sequence resulted in 280 operational taxonomic units (OTUs) following exclusion of rare OTUs (present at < 0.05% in all four countries). The subgingival plaque from dog populations located in the UK, USA, China and Thailand had a similar composition although the abundance of certain taxa varied significantly among geographical locations. Exploration of the effect of clinical status and age revealed a marked similarity among the bacteria associated with increased age and those associated with gingivitis: Young dogs and those with no gingivitis were dominated by taxa from the phyla Bacteroidetes and Proteobacteria whereas older dogs and those with moderate gingivitis were dominated by members of the Firmicutes. The plaque microbiota of small breed dogs was found to significantly differ to medium and large breeds and was dominated by species belonging to the Firmicutes.

**Conclusions:**

The bacterial associations with health, gingivitis and periodontitis were conserved across dogs from the UK, USA, China and Thailand. These bacterial signatures of periodontal health and disease have potential as biomarkers for disease detection.

**Supplementary Information:**

The online version contains supplementary material available at 10.1186/s12917-020-02660-5.

## Background

Periodontal disease is the most common oral disease of dogs worldwide [1–3]. Clinically the first sign is red and inflamed gums, gingivitis, which can progress into periodontitis where the ligaments and bones that support the tooth are progressively destroyed [4]. The initial stages of the disease can be managed with early identification and intervention, however, without effective treatment the chronic inflammation and tissue damage is likely to cause significant pain and tooth loss in the latter stages of the disease.

The disease results from a complex interplay between plaque bacteria, the host and environmental factors. Early culture-based studies to understand the role of bacteria in periodontal disease highlighted that canine bacterial species were different to those of humans [5–7]. This finding was supported by a subsequent molecular study which identified 353 taxa of which 80% were novel and only 16.4% were shared with the human oral microbiota [8]. Recently, advances in sequencing technologies coupled with new bioinformatics developments have enabled studies that have significantly enhanced our understanding of the canine bacterial species associated with health and periodontal disease [9–11].

Although the importance of the oral microbiota for canine health and disease is increasingly recognised, to the best of our knowledge there have been no studies to explore the variability in the composition of the canine oral microbiota across different geographies. Several studies of human subjects have shown that the mean proportions of plaque-associated species differ by country [12–14] and that there are oral microbial signatures capable of differentiating between ethnicities and between populations living in different geographical locations [15–19]. Furthermore, studies of isolated communities of people have resulted in identification of a number of novel taxa not previously described, resulting in a distinctive composition of the oral microbiota [20, 21]. In contrast, other investigators have concluded that the oral microbiota was not associated with the geographic regions studied [22, 23].

The aim of this study was to investigate associations between the canine oral microbiota and geographical location in a cross-sectional study of dogs from the United Kingdom (UK), United States of America (USA), China and Thailand. The influence of periodontal health status, age and breed size were also taken into consideration as it was not possible to control for the cohorts of dogs presenting at the veterinary clinics. The hypothesis was that bacterial associations with periodontal health and disease will be conserved across individuals irrespective of the country in which they reside.

## Results

### Study cohort

Subgingival plaque samples from 587 dogs were included in the study. Breed size and age are potential risk factors for periodontitis and therefore sample associated metadata were also obtained (Table 1). Due to missing data, there were ten dogs of unknown age from China, four dogs of unknown breed size and one dog of unknown sex from the UK. The cohort of dogs from Thailand had a significantly lower age than dogs from the other three geographical locations (*p* < 0.001). Chi-squared analysis showed that there was a significant difference in the distribution of breed size across the dogs sampled in each of the four geographical locations (*p* < 0.001). There was no significant difference in the ratio of males to females (*p* = 0.971).

**Table 1:** Summary of metadata for each of the four locations

	UK	USA	China	Thailand
**Age (years)**				
Average	5.78	5.61	5.02	2.67
(95% CI)	(5.26, 6.3)^b^	(4.93, 6.29)^b^	(4.17, 5.87)^b^	(2.16, 3.18)^a^
Range	1.5 to 15	1 to 14.5	1 to 14	0.8 to 14
**Breed size**				
Small (<10 kg)	36 (17.6%)	56 (46.3%)	57 (42.5%)	22 (17.7%)
Medium (10-25 kg)	76 (36.9%)	24 (19.8%)	43 (32.1%)	81 (65.3%)
Large (>25 kg)	93 (45.1%)	41 (33.9%)	33 (24.8%)	21 (16.9%)
**Sex**				
Female	94 (45.2%)	54 (44.6%)	55 (45.1%)	59 (47.6%)
Male	114 (54.5%)	67 (54.5%)	67 (54.5%)	65 (52.4%)

^a, b^Significance with Tukey HSD homogenous groups at 5% within model

The sample collection procedure differed between the UK and the other three geographical locations (see methods) and therefore clinical status was determined on a whole mouth and on a teeth sampled basis. There were significant differences in the clinical status of dogs across the four locations as shown in Table 2. For example, the dogs from China had a higher average gingivitis score and a lower percentage of healthy teeth (G0) than the other geographical locations. Dogs from Thailand had a higher percentage of teeth with periodontitis and the percentage of missing teeth was lower in the UK dogs compared to dogs from China, Thailand and USA.

**Table 2:** Summary of clinical status of dogs across four locations

Model	Location			
	UK	USA	China	Thailand
**Average gingivitis score in mouth**	1.2 (1.05, 1.35)^a^	1.13 (0.95, 1.32)^a^	1.41 (1.29, 1.53)^b^	1.06 (0.92, 1.2)^a^
**Average gingivitis score sampled teeth**	1.35 (1.18, 1.51)^bc^	1.13 (0.95, 1.31)^ab^	1.41 (1.29, 1.52)^c^	1.06 (0.92, 1.19)^a^
**% Healthy teeth in mouth**	35.01 (28.82, 41.75)^a^	36.74 (28.55, 45.78)^a^	11.34 (6.9, 18.07)^b^	37.73 (29.55, 46.67)^a^
**% Healthy sampled teeth**	33.87 (25.44, 43.47)^a^	36.9 (29.65, 44.8)^a^	11.38 (7.39, 17.14)^b^	37.86 (30.63, 45.67)^a^
**% periodontitis teeth in mouth**	7.81 (6, 10.1)^b^	6.19 (4.17, 9.1)^b^	6.11 (4.17, 8.38)^b^	13.29 (10.26, 17.03)^a^
**% periodontitis teeth sampled**	10.99 (7.75, 15.35)^a^	5.78 (3.86, 8.58)^b^	5.74 (3.89, 8.38)^b^	13.03 (10.08, 16.69)^a^
**% missing teeth in mouth**	2.24 (1.5, 3.35)^a^	3.66 (2.42, 5.5)^ab^	4.48 (3.14, 6.35)^b^	3.76 (2.52, 5.6)^ab^

^a, b, c^Significance with Tukey homogeneous groups at 5% within model

Further exploration of the correlations between the clinical status of the whole mouth compared to the sampled teeth showed that the USA, Thailand and China were almost identical due to nearly all the teeth in the mouth being sampled (supplementary figure 1). The exceptions were 33 dogs with >PD2 (periodontal disease stage 2) teeth in the mouth and two dogs with PD2 teeth in a “healthy mouth” that were not included in the sample. For the UK samples there was correlation with respect to health and gingivitis although there was less correlation than the other three geographical locations (supplementary figure 1A & 1B). However, with respect to periodontitis the correlation was poor, defined by the divergence as the number of periodontitis teeth sampled increased (supplementary figure 1C). This occurs due to the fact that in most instances the UK dogs only had 6–8 periodontitis teeth sampled and no other teeth in the mouth included.

### Sequence quality

Analysis of the 587 subgingival plaque samples by 454-pyrosequencing of the 3′ end of the V1-V3 region of the 16S rDNA gene resulted in the generation of 10,537,181 sequence reads that passed the sequencing providers initial sequence quality filter. After AmpliconNoise filtering 6,944,757 sequence reads remained. The number of sequences per sample ranged from 2027 to 30,050. The mean number of sequences per sample was 13,868 with 95% confidence interval (12,912, 14,825), 12,016 (11,321, 12,710), 8807 (8110, 9503) and 11,460 (10,944, 11,976) for UK, USA, China and Thailand respectively. Generalised linear model (GLM) analysis showed that the UK sequence depth was more variable than the other countries and had a significantly higher sequence depth on average than all other geographical locations (*p* < 0.001). China had a lower sequence depth on average than all other geographical locations (*p* < 0.001).

### Bacterial composition of canine plaque

A total of 13,530 OTUs were identified by clustering at ≥98% sequence identity and of these 13,250 were rare; 7819 OTUs had less than two counts across all geographical locations and 5431 OTUs had no geographical location with > 0.05% sequence reads. The percentage of these rare sequences did not significantly differ between geographical locations with mean values of 3.64% (3.22, 4.11), 3.13% (2.60, 3.77), 3.56 (2.92, 4.31) and 3.09 (2.55, 3.73) in UK, USA, China and Thailand respectively (*p* > 0.05). After exclusion of the rare OTUs a total of 280 OTUs remained.

Of the 280 OTUs, 254 (91%) shared ≥98% sequence identity and ≥ 98% coverage to sequences within the Silva database. Of these 140 (50%) mapped to sequences from a previous study of the canine oral microbiota (Dewhirst et al., 2012; Genbank accession numbers JN713151-JN713566 & KF030193-KF030235). Thirty (11%) and 71 (25%) mapped to sequences annotated to the species and genus level respectively and the remaining 14 (5%) had only been annotated to the family, order or phylum level. For the remaining 25 (9%) OTUs the percentage identity or coverage was less than 98% but all had ≥95% sequence identity to sequences within the Silva database and ≥ 86.6% coverage.

**Fig. 1:**
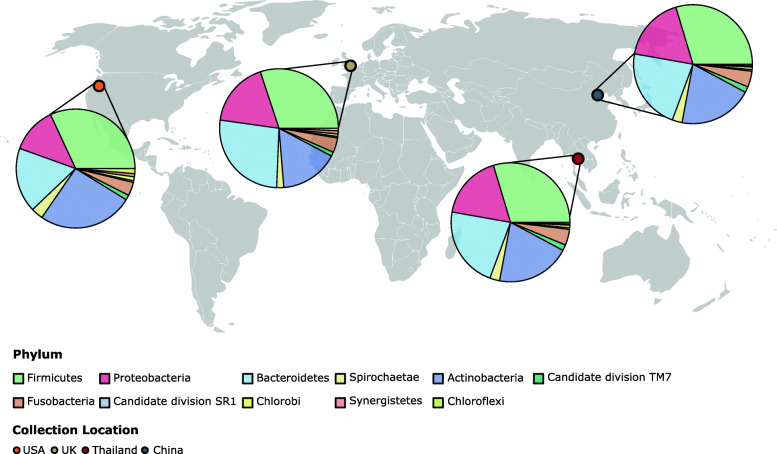
Map showing the geographical locations where canine subgingival plaque samples were collected. Pie charts depict the average relative abundance of each phyla in samples from USA, UK, Thailand and China (left to right). Map design by www.123Freevectors.com

Overall eleven phyla were identified and the proportions of each were similar across all four geographical locations (Fig. 1). Firmicutes were the most abundant on average across all samples (29.4%) followed by Actinobacteria (22.5%), Bacteroidetes (19.8%), Proteobacteria (14.9%), Fusobacteria (3.6%), Spirochaetes (2.5%), TM7 (1.5%), Chloroflexi (0.76%), Synergistetes (0.63%), Chlorobi (0.63%) and SR1 (0.36%).

At ≥98% sequence identity, which approximates to species level, *Porphyromonas cangingivalis* was the most dominant taxa with an average abundance of 5.7% across all samples. A further 23 OTUs had average relative abundances > 1% and together accounted for 39.4% of sequence reads (Table 3). Of the remaining OTUs, 128 were present with average proportions ranging from 0.1 to 1% and these accounted for 45.7% of the sequence reads, and 128 OTUs had average proportions < 0.1% and together these accounted for 6.8% of the sequence reads.

**Table 3:** Operational taxonomic units with an average proportion > 1% in canine subgingival plaque from dogs with healthy gingiva, gingivitis and mild periodontitis (all locations)

Taxa	Proportion of total sequence reads
*Porphyromonas cangingivalis*	5.67%
Novel *Actinomyces*	5.08%
Novel *Moraxella*	2.95%
Peptostreptococcaceae bacterium COT-077	2.55%
*Actinomyces* sp. COT-083	2.51%
Novel *Bergeyella*	2.00%
Clostridiales bacterium COT-028	1.86%
*Actinomyces* sp. COT-252	1.69%
Novel *Fusobacterium*	1.59%
*Filifactor villosus*	1.59%
Novel *Porphyromonas*	1.52%
Novel *Peptostreptococcus*	1.46%
Novel *Euzebya*	1.39%
Novel *Corynebacterium*	1.35%
Peptostreptococcaceae bacterium COT-004	1.32%
*Neisseria shayeganii*	1.30%
Novel *Porphyromonas*	1.30%
Erysipelotrichaceae bacterium COT-311	1.29%
*Granulicatella* sp. COT-095	1.19%
*Porphyromonas gulae*	1.14%
Novel *Johnsonella*	1.11%
*Porphyromonas canoris*	1.11%
Novel *Corynebacterium*	1.09%
Novel *Euzebya*	1.04%

### Composition of subgingival plaque

The composition of subgingival plaque was investigated and clearly showed evidence of a core microbiota in canine subgingival plaque based on presence of OTUs (presence was defined as > 0.05%); that is a set of common OTUs across all dogs and geographical locations (supplementary figure 2). The OTUs formed three main clusters of similarly present OTUs which together accounted for 46.6% of the total sequences (supplementary figure 2). Cluster A contains 10 OTUs (3.6% of the total number of OTUs), cluster B contains 16 OTUs (5.7%) and cluster C comprises 50 OTUs (17.9%).

For cluster A, the percentage of samples in which the OTU was present ranged from 4.9 to 84.7% with all but 2 OTUs present in more than 65% of samples. The OTUs were distributed across two phyla; Proteobacteria (6 OTUs) and Bacteroidetes (4 OTUs). The most abundant taxa were *Neisseria shayeganii* (1.3%) and novel, as yet unclassified, species from the genera *Bergeyella* (2.0%) and *Moraxella* (3.0%). The percentage of samples positive for the OTUs in cluster B ranged from 17 to 94.5% with the majority occuring in more than 78% of samples. The most represented phyla were Firmicutes (6 OTUs), Bacteroidetes (3 OTUs) and Actinobacteria (4 OTUs). Five OTUs were present in > 90% of the samples; two species belonged to the genus *Actinomyces* (*Actinomyces* sp. COT-252, *Actinomyces* sp. COT-083) and the other species were a novel unclassified species belonging to the genus *Fusobacterium*, TM7 phylum sp. COT-305 and Peptostreptococcaceae bacterium COT-004. A further four OTUs were present in greater than 80% of samples and had an average relative abundance of > 1%; Erysipelotrichaceae bacterium COT-311, *Filifactor villosus and *, novel species from the genera *Euzebya* and *Johnsonella*. With respect to cluster C the percentage of samples in which the OTUs were present ranged from 3.1 to 91.3% with the majority (70%) present in more than 50% of samples. One OTU, a novel unclassified species belonging to the genus *Fusobacterium* was present in >90% of samples. Seven OTUs within this cluster were present at > 1% and present in at least 50% of samples: *Porphyromonas canoris*, Clostridiales bacterium COT-028, *Porphyromonas gulae*, *Granulicatella* sp. COT-095 and novel species from the genera *Corynebacterium *and *Euzebya*.

There were only nine OTUs that were not present in all four countries. Five OTUs were absent in plaque collected from dogs in Thailand but present in dogs from the other three geographical locations: *Actinomyces* sp. COT-412 was present at > 0.07% in USA, UK and China, a further three OTUs (novel species from the genera *Murdochiella* and *Actinomyces* and a novel species from the family Christensenellaceae) were present at < 0.03% in USA and UK but > 0.05% in China, *Escherichia-Shigella* was prevalent in the UK dogs (> 0.067%) but present at < 0.0004% in China and the USA. *Streptococcus orisasini* (prevalence 0.044%) was only present in dogs from the UK and completely absent from dogs in the USA, China and Thailand and another novel *Streptococcus* species was present at 0.056% in the UK dogs and present at < 0.02% in China and Thailand but absent from the USA dogs. Another novel *Streptococcus* species was present at > 0.1% in USA dogs but was absent from dogs located in Thailand and China and present at < 0.006% in UK dogs. Finally, a novel *Actinomyces* species was present at high abundance in dogs from Thailand (> 0.06%) and UK dogs and present at < 0.015% in dogs from China but absent from USA.

### Effect of geographical location, clinical status, age and dog breed size on the plaque microbiota

**Fig. 2:**
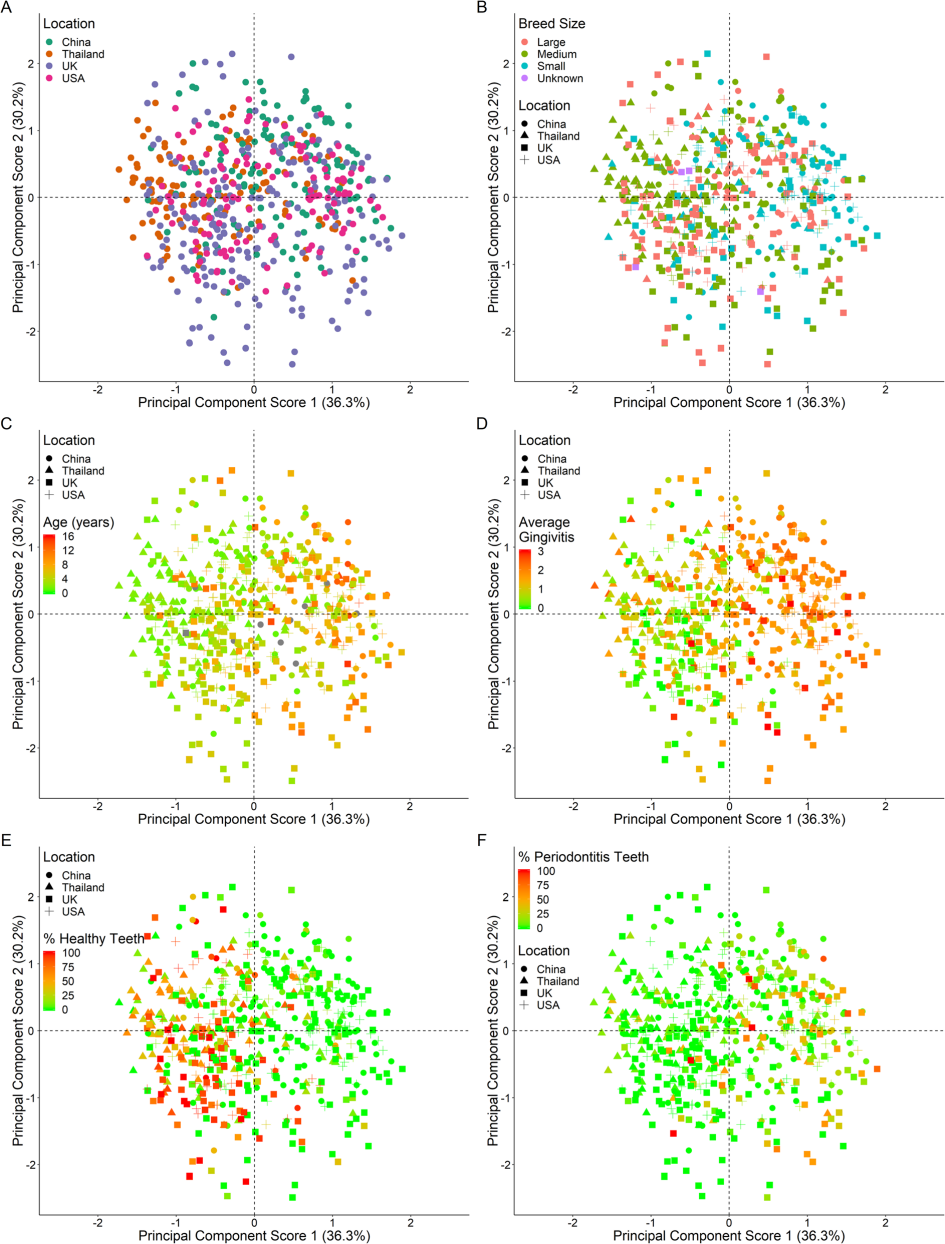
Principal component scores 1 versus 2 of OTUs coloured by **a**) geographical location, **b**) breed size, **c**) age **d**) average gingivitis, **e**) percentage healthy teeth and **f**) percentage periodontitis teeth. **b**) to **f**) are also shaped by geographical location

An exploratory principal components analysis (PCA) showed no discrete clustering of plaque microbiota by geographical location; the variance was possibly larger in the UK, although the number of dogs sampled was greatest in this geographical location (Fig. 2a). No discrete clustering was seen by breed size (Fig. 2b), sex or neuter status (data not shown). However, progression was evident in component one with increasing age suggesting that the variance in the OTUs changes with advancing age (Fig. 2c). Likewise, clinical status showed progression in component one suggesting the variance changes with percentage healthy teeth and average gingivitis (Fig. 2d and e). Progression was also evident with respect to the number of teeth with periodontitis although the pattern was not as clear (Fig. 2f). This indicates that the composition of the plaque microbiota is influenced by the extent of gingivitis and periodontitis. Similar patterns were evident for both the mouth and sampled teeth with respect to percentage of healthy teeth, average gingivitis and percentage of periodontitis teeth suggesting that clinical status of the mouth and the teeth sampled are highly correlated (data not shown). Therefore, only the sampled teeth data were investigated further to identify bacterial associations with geographical location, age, clinical status and breed size.

Of the 280 OTUs analysed by GLM, 237 had significant effects and of these 14 had significant geographical location interactions and 28 had interactions with other covariates (gingivitis, age, breed size, percentage healthy teeth and percentage of periodontitis teeth).

### Effect of geographical location

Of the 14 significant interactions with geographical location, only six OTUs had an interaction between geographical location and clinical status and none with percentage healthy or periodontitis teeth, suggesting that bacterial associations with health or disease did not change by geographical location. There were also six interactions between geographical location and age and two with breed size (Supplementary table 1). Of the models without significant geographical location interactions, 152 OTUs had significant geographical location effects indicating that the relative abundance of bacteria significantly differed by geographical location (Supplementary table 2). Of the 912 pairwise comparisons, 355 were significant and the majority of these were due to differences between the UK and the other three countries (24.5% of OTUs significantly different between the UK and China, 20% significantly different between the UK and Thailand and 19.4% significantly different between the UK and USA). The next most different were Thailand and China (14.4% of pairwise comparisons significant), followed by the USA and China (11.3%) and then USA and Thailand (10.4%).

**Fig. 3:**
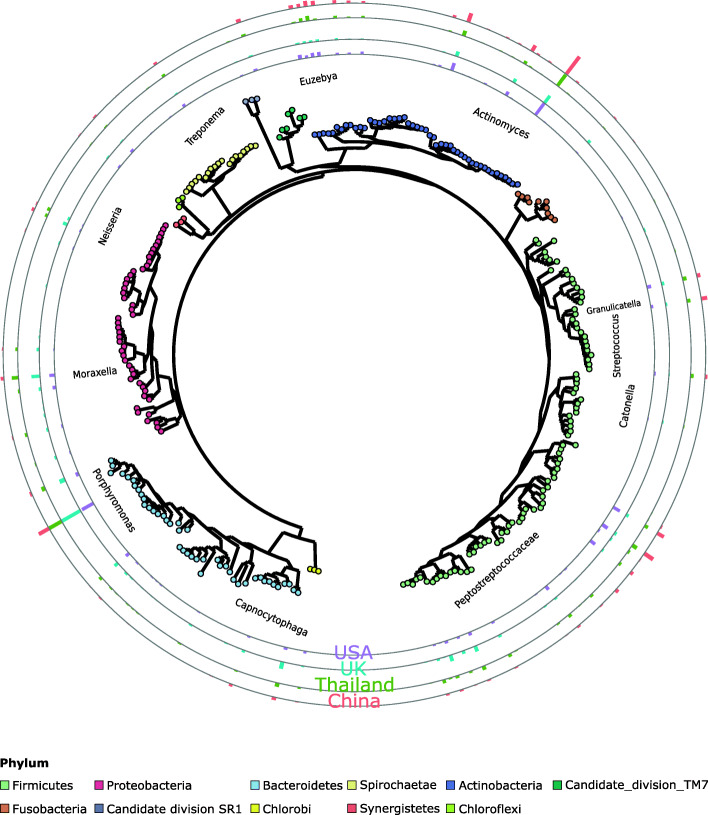
Phylogenetic tree of representative sequences for 280 OTUs. Nodes are coloured by best hit phylum classification, notable clades are highlighted in grey. Ring annotations represent mean relative abundances for the four countries surveyed; from out to in, China in red, Thailand in green, UK in blue and USA in purple. The 153 OTUs with geographic significant effects have coloured ring annotations, whereas non-significant OTUs ring annotations are grey

The UK was characterised by significantly higher estimated mean proportions of OTUs from the phyla Bacteroidetes and Proteobacteria and significantly lower estimated proportions of OTUs from the phyla Actinobacteria compared to the USA, Thailand and China (Supplementary table 2, Fig. 3). A number of OTUs belonging to the Firmicutes were also significantly less abundant in the UK compared to the other countries but other OTUs belonging to this phyla had significantly higher estimated abundances. Within the phylum Bacteroidetes the most abundant OTUs (> 0.3%) showing the biggest differences between the UK and the other three countries (odds ratio > 2) included two novel species from the genera *Bergeyella* and *Porphyromonas*. Within the phylum Proteobacteria the most notable differences were with respect to the species *Cardiobacterium* sp. COT-177, *Desulfomicrobium orale* and *Pasteurella dagmatis* which were all significantly more abundant in the UK compared to all other countries. With respect to the Firmicutes, two novel species from the genera *Peptococcus* and *Peptostreptococcus* and members of the family Peptostreptococcaceae were significantly more abundant in the UK compared to Thailand, China and USA. Members of the Actinobacteria that had a significantly lower estimated proportion in the UK compared to the other countries included two novel species from the genus *Euzebya* and two species from the genus *Actinomyces* (*A. weissii* and a novel species). With respect to the Firmicutes that had a significantly lower estimated abundance in the UK compared to other countries none were common to all three countries. One other phyla worthy of mention is Spirochaetes where one species, *Treponema denticola*, was significantly lower in the UK population compared to the other three countries.

Dogs from the USA were characterised by having higher estimated mean proportions of OTUs from the phylum Firmicutes compared to China and Thailand (Supplementary Table 2). Of the most abundant OTUs (> 0.3%) with the biggest difference between countries (odds ratio > 2) only one species of Firmicutes, *Helcococcus* sp*.* COT-069 had a significantly higher proportion in the USA compared to Thailand and China. Plaque collected from dogs in the USA also had higher estimated proportions of Actinobacteria and Proteobacteria compared to Thailand and higher estimated proportions of OTUs from the phyla Bacteroidetes compared to China. The USA had lower estimated proportions of OTUs from the phyla Proteobacteria compared to Thailand and lower estimated proportions of OTUs from the phyla Actinobacteria than China.

In terms of the OTUs that differed in their mean relative abundance between Thailand and China, the phyla Firmicutes, Proteobacteria and Bacteroidetes had higher estimated proportion in Thailand compared to China. The most abundant (> 0.3%) Firmicutes with the biggest difference between the two countries (odds ratio > 2) were *Frigovirgula* sp*.* COT-007, *Tissierella* sp*.* COT-029, a novel species from each of the genera *Catonella* and *Parvimonas*, a novel species from the family Erysipelotrichaceae and four species from the family Peptostreptococcaceae. With respect to the Proteobacteria, *Campylobacter* sp*.* COT-011 and two species form the genus *Moraxella* (COT-018 and a novel species) were the most notable. In terms of the phylum Bacteroidetes, *Bacteroides* sp*.* COT-040, *Porphyromonas gulae* and *Prevotella* sp*.* COT-226 had higher estimated proportions in Thailand compared to China. A number of OTUs from the phylum Actinobacteria were significantly less abundant in Thailand compared to China, for example, Actinobacteria bacterium COT-376, *Actinomyces* sp*.* COT-252, *Propionibacterium* sp*.* COT-296, a novel species from the genus *Actinomyces* and two novel species from the genus *Corynebacterium*.

### Associations with health, gingivitis and periodontitis

Of the 15 interactions with average gingivitis, eight were with percentage of teeth with periodontitis and three with the percentage of healthy teeth (Supplementary Table 1). When none of the sampled teeth had periodontitis there were three OTUs that had significantly higher estimated mean proportions when moderate gingivitis compared to no gingivitis and all belonged to the phylum Firmicutes (Peptostreptococcaceae bacterium COT-068, a novel species belonging to the genus *Peptostreptococcus* and Clostridiales bacterium COT-027). When all of the sampled teeth had periodontitis there were five OTUs that had significantly higher estimated proportions with moderate gingivitis compared to no gingivitis and these belonged to the phyla Proteobacteria (*Campylobacter* sp*.* COT-011), Bacteroidetes (*Bacteroides* sp*.* COT-040 and a novel species from the genus *Alloprevotella*) and Firmicutes (*Frigovirgula* sp*.* COT-007 and a novel species from the genus *Fusobacter*). With respect to the interaction between gingivitis and percentage healthy teeth, there were two OTUs, belonging to the phyla Actinobacteria (a novel species from the genus *Actinomyces*) and Firmicutes (Peptostreptococcaceae bacterium COT-030), that had significantly higher estimated mean proportions with moderate compared to no gingivitis when all the teeth had periodontitis. In contrast, there was one OTU belonging to the Proteobacteria (a novel species from the genus *Haemophilus*) that had significantly higher predicted proportions with moderate compared to no gingivitis when none of the teeth had periodontitis. There were also three OTUs with an interaction between gingivitis and breed size and one with a significant interaction between gingivitis and age (Supplementary Table 1).

**Fig. 4:**
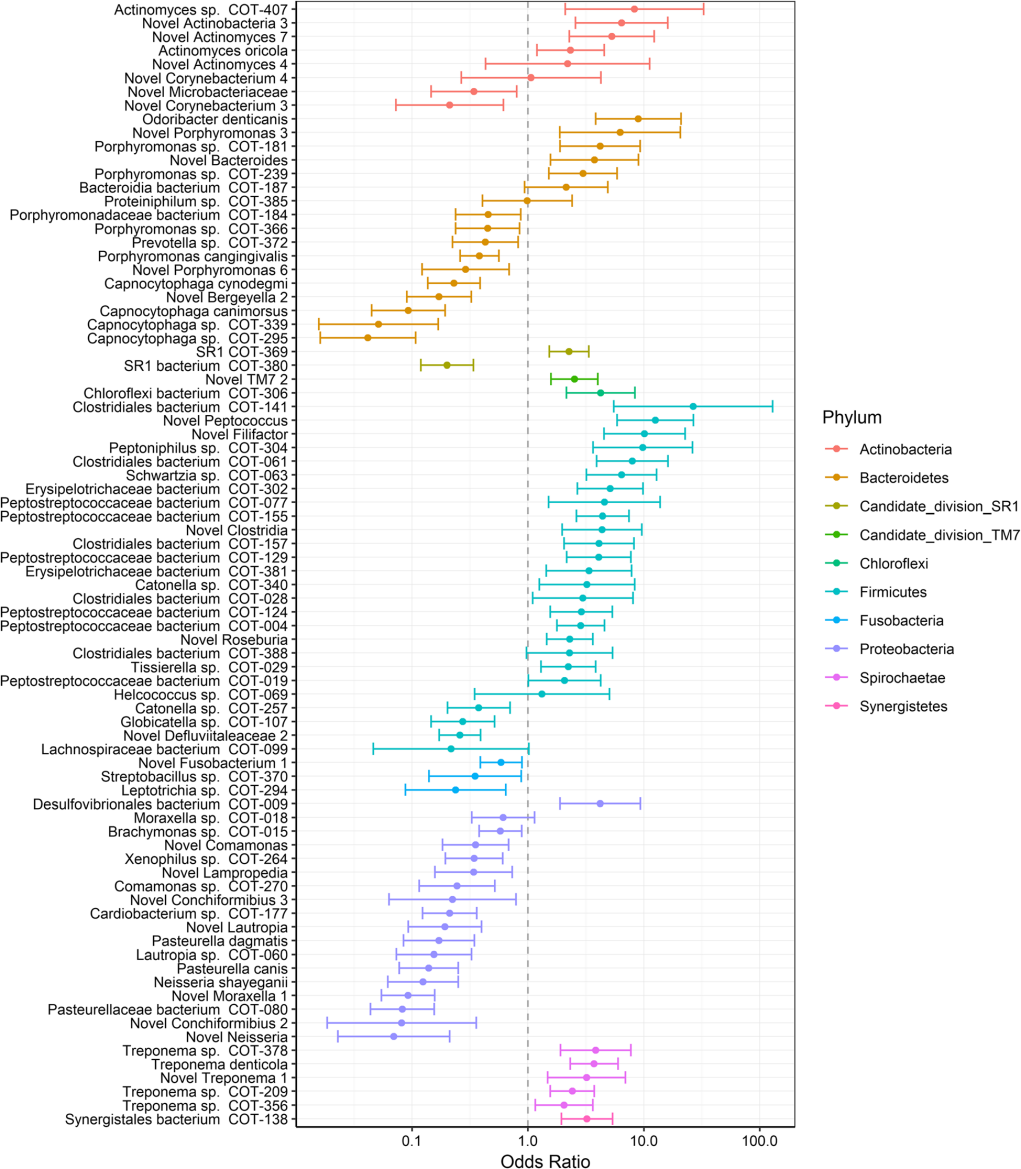
Odds ratios for OTUs with significant gingivitis effects: No gingivitis (G0) compared to moderate gingivitis (G3). Bars depict 95% confidence intervals and colours represent the phylum

Of the 82 OTUs found to have a significant average gingivitis effect, 39 had a significantly higher estimated mean proportion at a gingivitis score of three compared to a score of zero and 35 had a significantly higher proportion at a gingivitis score of zero compared to a score of three (Supplementary table 2, Fig. 4). Of the OTUs associated with moderate gingivitis the majority belonged to the phylum Firmicutes (20 OTUs), followed by Bacteroidetes (5 OTUs), Spirochaetes (5 OTUs) and Actionbacteria (4 OTUs). The remaining five OTUs belonged to the phyla SR1, TM7, Chloroflexi, Proteobacteria and Synergistetes. The most dominant members of the Firmicutes (> 0.3%) with odd ratios greater than two were; three species from the Peptostreptococcaceae family (COT-019, COT-077 and COT-004) two species from the class Clostridiales (COT-028 and COT-141), *Tissierella* sp. COT-029, Erysipelotrichaceae bacterium COT-302, *Schwartzia* sp*.* COT-063 and three novel species from the genera *Filifactor*, *Peptococcus* and *Roseburia*. With respect to the other phyla the most abundant OTUs (> 0.3%) that were strongly associated with gingivitis (odds ratio > 2) were *Porphyromonas* sp. COT-239 and a novel *Porphyromonas* species which belong to the phyla Bacteroidetes, *Treponema denticola* a member of the Spirochaetes and *Actinomyces* sp*.* COT-407 and a novel species belonging to the phylum Actinobacteria.

Of the 35 OTUs that were at a significantly higher mean proportion at a gingivitis score of zero compared to a score of three, these predominantly belonged to two phyla Proteobacteria (16 OTUs) and Bacteroidetes (10 OTUs) with the remaining nine OTUs belonging to the phyla Firmicutes, Fusobacteria, Actinobacteria and SR1. The most abundant OTUs (> 0.3%) from the phylum Actinobacteria with an odds ratio > 2 were Pasteurellaceae bacterium COT-080, *Neisseria shayeganii*, *Cardiobacterium* sp*.* COT-177, *Xenophilus* sp. COT-264, *Pasteurella dagmatis*, two species of *Lautropia* (COT-060 and a novel species) and two novel species from the genera *Conchiformibius* and *Moraxella*. In the case of the Bacteroidetes the most abundant OTUs with an odds ratio > 2 were four species of *Capnocytophaga* (*C. canimorsus*, *C. cynodegmi* COT-295 and COT-339), *Porphyromonas cangingivalis*, Porphyromonadaceae bacterium COT-184 and a novel species from the genus *Bergeyella*.

Of the 33 OTUs found to have a significant percentage healthy teeth effect (0% versus 100% healthy teeth) only ten were present at relatively high abundance (> 0.3%) and had an odds ratio > 2. Four increased with increasing proportion of healthy teeth; a novel species from the genus *Neisseria*, *Conchiformibius steedae*, a novel *Peptococcus* species and a novel species from the family Pasteurellaceae. Six decreased with increasing proportion of healthy teeth; three species form the Peptostreptococcaceae family, *Helococcus* sp*.* COT-069, Actinobacteria bacterium COT-376 and a novel species from the genus *Corynebacterium*.

Of the 10 OTUs found to have a significant percentage teeth with periodontitis effect (0% versus 100%) only three were present at relatively high abundance (> 0.3%) and had an odds ratio > 2; a novel species from the genus *Actinomyces*, *Treponema* sp*.* COT-356 and Peptostreptococcaceae bacterium COT-030.

### Effect of age

**Fig. 5:**
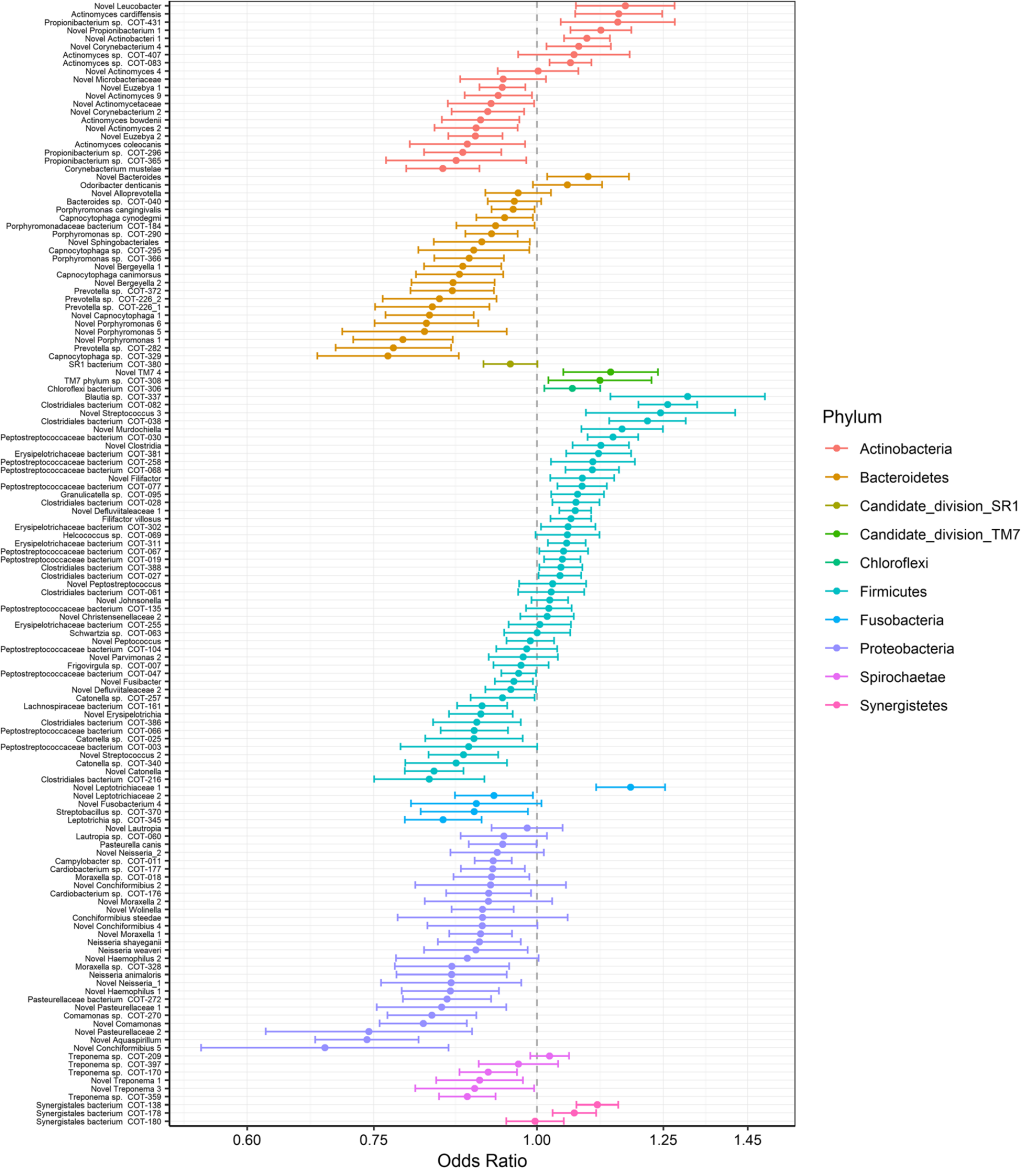
Odds ratios for OTUs with significant age effects. Age 15 years compared to age one. Bars depict 95% confidence intervals and colours represent the phylum

There were seven OTUs that had an interaction between age and clinical status or age and breed size (Supplementary table 1). Of the 138 OTUs without interactions that were found to have significant age effects, 36 had a significantly higher estimated mean proportion at age 15 than age 1 and 70 had a significantly lower proportion at age 15 compared to age 1. Of those that were significantly more abundant at an older age, the majority belonged to the phyla Firmicutes (22 OTUs) and Actinobacteria (7 OTUs). The remaining seven OTUs were members of the phyla Bacteroidetes, Synergistetes, TM7, Chloroflexi and Fusobacteria (Supplementary table 2, Fig. 5). Within the phylum Firmicutes there were 12 abundant OTUs (> 0.3% of population) that had an odds ratio > 2 when comparing the estimated proportion at age 15 to age one: Four were assigned to the family Peptostreptococcaceae, two to the family Erysipelotrichaceae, two to the class Clostridiales and four could be assigned to the genus or species level (*Blautia* sp*.* COT-337, *Granulicatella* sp*.* COT-095, *Filifactor villosus* and a novel species belonging to the genus *Streptococcus*). With respect to members of the phylum Actinobacteria the four most abundant OTUs (> 0.3%) with an odds ratio greater than two were *Actinomyces* sp*.* COT-083, *Propionibacterium* sp*.* COT-431 and two novel species one from the genus *Corynebacterium* and the other from the genus *Leucobacter*. Of the 70 OTUs that had a significantly lower proportion at age 15 compared to age 1, the majority belonged to four phyla; Proteobacteria (20 OTUs), Bacteroidetes (19 OTUs), Firmicutes (13 OTUs) and Actinobacteria (11 OTUs). The remaining 11 OTUs belonged to the phyla Fusobacteria and Spirochaetes. With respect to the Proteobacteria phylum the most abundant members (> 0.3%) with the biggest difference between ages 15 and one (odds ratio > 2) were three species of *Neisseria* (*N. animolaris, N. shayeganii, N. weaveri*), two species from the genus *Moraxella* (*Moraxella* sp. COT-018 and a novel species), two novel species from the family Pasteurellaceae, *Campylobacter* sp*.* COT-011 and a novel species from the genus *Aquaspirillum*. Representing the phylum Bacteroidetes there were two species from the genus *Capnocytophaga* (*C. canimorsus, C. cynodegmi*), a novel species from the genus *Bergeyella*, *Prevotella* sp. COT-226, Porphyromonadaceae bacterium COT-184 and two species from the genus *Porphyromonas* (COT-290 and a novel species). In the phylum Firmicutes there were two novel species, one from the genus *Catonella* and one from the genus *Streptococcus*. With respect to the phylum Actinobacteria there were two species from the genus *Corynebacterium* (*C. mustelae* and a novel species), two novel species from the genus *Euzebya*, a novel *Actinomyces* species and *Propionibacterium* sp*.* COT-296.

### Effect of breed size

There were eleven OTUs that had an interaction with breed size, three with gingivitis, four with the percentage of periodontitis teeth, two with the percentage of healthy teeth and two with age (Supplementary table 1). Of the 67 OTUs found to have a significant breed size effect, the majority of these (~ 90%) were due to small breeds significantly differing to medium and large breeds (Supplementary table 2). Small breeds of dog generally had higher estimated mean proportions of OTUs from the phylum Actinobacteria and lower estimated proportions of OTUs from the phyla Bacteroidetes and Proteobacteria than medium and large dogs. The phylum Firmicutes was present at higher and lower estimated proportions in small breed dogs compared to medium and large breeds. Despite the gross phylum level differences only six OTUs were present at relatively high abundance (> 0.3% of total population) and had an odds ratio > 2.

### Species diversity

**Fig. 6:**
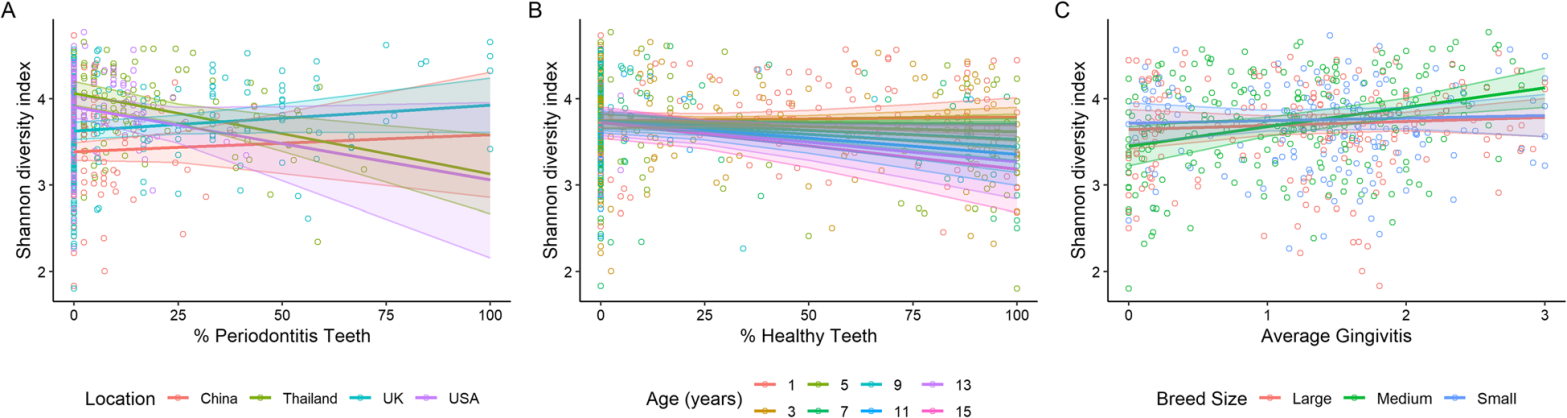
Shannon diversity indices by A) percentage of teeth with periodontitis and geographical location with raw data represented by open circles coloured by geographical location, B) percentage healthy teeth and age in years with raw data shown as open circles coloured by age and C) average gingivitis score and breed size with raw data represented as open circles coloured by breed size. The average relationships are shown as solid lines with shaded 95% confidence intervals

Linear regression analysis of the Shannon diversity indices found significant interactions of geographical location with percentage of periodontitis teeth (*p* = 0.0003). The percentage of periodontitis teeth with geographical location interaction was due to dogs from Thailand having a significantly higher mean Shannon diversity index at 0 % periodontitis teeth but lower indices at 100% periodontitis teeth than dogs from the other geographical locations (*p* = 0.003; Fig. 6a). The mean Shannon diversity index was 4.07 (3.93, 4.20) when none of the sampled teeth had periodontitis reducing to 3.13 (2.67, 3.59) when 100% of the sampled teeth had periodontitis which is a difference of 0.94 (0.25, 1.62). The Shannon diversity index was not significantly correlated with the percentage of periodontitis teeth with respect to dogs from UK, USA and China (*p* = 0.266, *p* = 0.288 and *p* = 0.976 respectively).

Linear regression analysis of the Shannon diversity indices also identified significant interactions of age with percentage healthy teeth (*p* = 0.038). The Shannon diversity index decreased with age and the difference in means between ages one and 15 increased with increasing percentage of healthy teeth (Fig. 6b): When no healthy teeth were sampled the difference in means was 0.02 (− 0.24, 0.29) whereas when all the sampled teeth were healthy the difference was 0.61 (− 0.06, 1.28). However, none of these differences in means were significant *p* = 0.949 for 0 % healthy teeth and *p* = 0.084 for 100% healthy teeth.

Linear regression analysis of the Shannon diversity indices also found significant interactions of average gingivitis score with breed size (*p* = 0.005). The average gingivitis score with breed size interaction was due to medium dogs having a significantly lower mean Shannon diversity index at a gingivitis score of zero but higher indices on average at a gingivitis score of 3 (*p* = 0.005; Fig. 6c). The mean Shannon diversity index was 3.45 (3.22, 3.68) at a gingivitis score of zero but increased to 4.13 (3.9, 4.35) at a gingivitis score of three which is a difference of − 0.68 (− 1.18, − 0.17). There was no correlation between Shannon diversity index and gingivitis score for small and large breed dogs (*p* = 0.949 and *p* = 0.832 respectively).

## Discussion

This is the largest study to date of the canine oral microbiota; it describes the bacterial composition of subgingival plaque samples from 587 dogs residing in four countries (UK, USA, China and Thailand), determined by 454-pyrosequencing. A major strength of the study is that the analysis of all plaque samples was performed in the same laboratory, eliminating the possibility of technical differences in microbial enumeration. A potential weakness however, is that although dogs were selected using the same inclusion and exclusion criteria there were differences in age, breed, breed size and clinical status across countries. This was unavoidable as it was not possible to control for the breeds, sizes and ages of dogs presenting at the veterinary hospitals. To minimise any ethical impact of the study no animals were given a general anaesthesia solely for the purpose of this trial. This was a stipulation of the ethical review body. This consideration did mean that to recruit significant numbers of animals in a reasonable time frame the breed, breed size and age factors were variable. The imbalance in groups was, therefore, unfortunately unavoidable. Breed size and age were therefore included in the statistical models to account for these factors alongside geographical location and periodontal status effects. This also accounted for the fact that periodontal health status, breeds size and age are potentially not independent variables. This ensured that any significant effect on the abundance of an OTU with periodontal health state, for example, was irrespective of the other confounding factor. Likewise, it also enabled the identification of interactions between variables. Another limitation was that plaque sampling methods were different between the UK and the other three geographical locations. In addition, scoring and sampling was undertaken by different individuals at the various geographical locations which could have led to bias. However, all scorers received training by the same Diplomate in Veterinary Dentistry and no discrete clustering of samples by geographical location was observed indicating that different sampling protocols had little effect on the overall plaque microbiota composition. Although, intraoral dental radiographs should be taken as part of a comprehensive periodontal assessment this was not always possible as several of the veterinary hospitals did not have this capability due to cost and availability of the equipment within some of the countries. Scoring of periodontal disease was therefore based on clinical attachment loss, on one or more aspects of the tooth, as measured using a periodontal probe. The probing depths were based on the average estimated root length of a small, medium and large breed of dog and therefore may not be accurate for every tooth in the mouth. The stages of periodontal disease defined by the American Veterinary Dental College are merely descriptive and, for this reason, the scoring criteria used in this study were developed in collaboration with a diplomate of the European Veterinary Dental College (EVDC). The criteria defined were verified using dental radiographs from some of the dogs.

Overall the bacterial composition of subgingival plaque was comparable to that which has been reported previously [5, 8, 9, 11, 24]. For example, Firmicutes were the most abundant phyla on average across all samples (29.4%) followed by Bacteroidetes (19.8%) and Proteobacteria (14.9%). Furthermore, the species *Porphyromonas cangingivalis* dominated with an average abundance of 5.7% across all samples. Exploration of the effect of geographical location, clinical status, age and breed size on the microbiota revealed that the community composition was most influenced by geographical location, age and gingivitis.

There was a striking resemblance between the bacterial associations with age and gingivitis. The composition of plaque from young dogs and those with no gingivitis was dominated by taxa from the phyla Bacteroidetes and Proteobacteria. Representatives from the phylum Bacteroidetes included species belonging to the genera *Capnocytophaga* and *Bergeyella* along with Porphyromonadaceae COT-184. With respect to Proteobacteria species from the genera *Neisseria* (in particular *N. shayeganii*), *Moraxella* and the family Pasteurellaceae were examples of species common to young dogs and those without gingivitis. In contrast, the plaque microbiota of older dogs and those with moderate gingivitis was dominated by members of the Firmicutes. Representatives of this phylum included members of the genus *Filifactor*, the class Clostridiales (in particular Clostridiales bacterium COT-028) and the families Erysipelotrichaceae and Peptostreptococcaceae. The bacterial associations with gingivitis concur with findings from other studies [9, 11]. Furthermore, bacterial species belonging to the phylum Firmicutes have been shown to increase in relative abundance as the severity of gingivitis worsens [11]. This is the first study to investigate the effect of age on the canine oral microbiota.

This study showed evidence of a core microbiota in canine subgingival plaque across the UK, USA, China and Thailand; there were three clusters of species which together accounted for about a third of the OTUs and nearly 50% of the sequences. Furthermore, only about 3% of the taxa were not shared across geographical locations and the percentage of rare taxa did not significantly differ between countries. However, in terms of relative abundance over 50% of the OTUs differed across geographical locations and the majority of these were due to differences between the UK and the other three countries. This concurs with human studies in that no significant geographic patterning was observed with respect to the human salivary microbiome but specific genera did vary significantly in frequency among geographical locations [23]. Likewise, DNA-DNA hybridisation studies found that on average all species were detected in subgingival plaque samples from different countries but the proportions of a number of species differed [15, 16]. This suggests that diet and the environment do not significantly influence the composition of the oral microbiome. However, a study of the saliva microbiome of human groups living under very different climatic conditions showed only limited support for a core microbiome at the OTU level across three continental regions [18].

The most striking observation was that plaque samples from the UK dogs were characterised by higher estimated proportions of species from the phyla Bacteroidetes and Proteobacteria compared to the USA, China and Thailand. The phyla Proteobacteria and Bacteroidetes are associated with young dogs and healthy gingiva suggesting that the UK dogs had microbial profiles similar to young dogs or dogs with low levels of gingivitis compared to other countries. The UK was also depicted by lower proportions of taxa belonging to the phylum Actinobacteria compared to the other three geographical locations and this phylum was also associated with young dogs and dogs with no gingivitis. This finding might be explained by differences in how plaque samples were collected in the UK where a subset of teeth were sampled compared to whole mouth collections in the other three geographical locations. Alternatively, it could be the result of genetics or other environmental factors such as feeding practices and oral care regimes that may exist. Another noticeable trend was that the dogs from China had lower estimated proportions of the phylum Bacteroidetes compared to the UK, USA and Thailand and lower estimated proportions of the phylum Proteobacteria than the UK and Thailand. This might be due to the fact that dogs from China had higher levels of gingivitis compared to Thailand and the USA and a lower percentage of healthy teeth than the other three geographical locations. Bacteroidetes and Proteobacteria were more represented in Thailand than China possibly because the dog population from Thailand was significantly younger by about 2 years. However, dogs from Thailand had a significantly higher proportion of teeth with periodontitis than the other three locations. Despite these differences only six OTUs were shown to have geographical location interactions with clinical status suggesting that bacterial associations with health and disease did not significantly differ by geographical location. Several studies of human subjects have shown that the mean proportions of plaque-associated species differ by country [12–14].

Approximately 25% of the OTUs were found to have a significant breed size effect and the majority of these were due to small breeds significantly differing to medium and large breeds. This study highlighted that small dogs had higher levels of many Firmicutes species than medium and large breeds, such as members of the family Erysipelotrichaceae and the class Clostridiales, which were also associated with moderate gingivitis. Furthermore they had significantly lower predicted proportions of many of the species belonging to the phylum Proteobacteria which were associated with healthy gingiva in this study. This is the first study to explore breed size differences in the oral microbiota and therefore, to the best of our knowledge, there are no published studies to which this data can be compared. It was not possible within this study to investigate the impact of breed due to over 90 breeds within the study cohort and hence low numbers of representatives for each breed. The link between the oral microbiota and breed size might explain why periodontitis is more prevalent in small breeds of dog compared to larger breeds [2, 3, 25]. These differences with respect to breed size might also explain some of the geographical location differences observed as the UK dog population had larger breed dogs, Thailand had more medium sized dogs and the USA had more small dogs than China and Thailand. Studies of ethnic groups in the USA reported differences in the prevalence of micro-organisms associated with human periodontitis between whites, Hispanics, and Asian-Americans [26], between African-Americans, Native Americans and Asians [27] and between African-American, Asian-American and Hispanics [17]. Furthermore, a recent study showed evidence of ethnicity-specific clustering of microbial communities in saliva and subgingival biofilms which appeared to be capable of discriminating between ethnicities [19]. Further investigations are required to determine if there is a breed-specific microbial signature.

## Conclusions

We have shown that subgingival plaque from dog populations located in the UK, USA, China and Thailand has a similar bacterial composition although the abundance of certain taxa significantly varies among geographical locations. These differences might be explained by genetics, diet, veterinary practices and oral hygiene procedures. Most important however, is that the bacterial associations with health, gingivitis and periodontitis did not significantly differ by geographical location. This means that it is plausible that the bacterial species within dental plaque can be used to classify dogs with periodontal disease, irrespective of where they reside, resulting in potential improvements in disease detection.

## Methods

### Sample population

The study cohorts comprised client-owned dogs presented at pet hospitals in the UK, USA, China and Thailand. With respect to the UK dog population the bacterial composition of their subgingival microbiota has been described previously [9]. The samples from the USA were collected from dogs visiting Banfield Pet Hospital^®^ in Portland between September 2012 and May 2013. The samples from China were collected from dogs visiting the Meillian Animal hospital in Beijing between March 2013 and July 2014. The samples from Thailand were collected from dogs visiting pet hospitals within Kasetsart University in Huahin and Bangkok between March 2013 and June 2014. An owner survey was completed for all dogs which included questions about the dog’s breed, age, sex, neuter status and breed size (small < 10 kg, medium 10–25 kg, large > 25 kg).

Purebred and crossbred dogs over 1 year of age were included in the study if they had not received corticosteroids, antibiotics or professional dental cleaning in the preceding 3 months. Dog breeds predisposed to developing periodontitis (Greyhounds, Yorkshire terrier, Maltese and toy/miniature poodles) [2, 3, 28] were excluded as this may be a different form of the disease. Dogs that had moderate or severe periodontitis (> 25% attachment loss [29]) were also excluded. This was defined as two indicator teeth (maxillary 3rd incisor, canine, 3rd and 4th premolar and mandibular canine, 4th premolar and 1st molar) or six teeth in the whole mouth at PD3 or above. Only dogs under general anaesthesia for routine treatment for non-periodontal complications were screened for inclusion in the study. No dogs were anaesthetised solely for the collection of plaque samples.

Prior to the start of the study a sample size calculation was performed using data from our previous UK cross-sectional survey [9]. The calculation assumed that the species diversity and variability in the relative abundance of bacterial species in the other three countries would be similar to that observed in the UK dog population. Based on the power calculation a sample size of 35 dogs per health state (health, gingivitis and mild periodontitis) was targeted. This would enable at least a 2-fold change in relative abundance between health states to be detected for bacterial species present at high abundance (> 2.68% of the total population), a 3-fold change for bacterial species present at medium abundance (> 0.37% of total population) and a 5-fold change for bacterial species present at low abundance (> 0.06% of total population) with a power of at least 80% using an overall significance test level of 5% that incorporates adjustments for multiple testing [30].

### Clinical assessment

Clinical assessments were performed by three to five veterinary nurses or veterinarians at each of the four collection sites. All received a minimum of 2 days training on scoring and recording periodontal disease from a Diplomate of the European Veterinary Dental College (Milella L).

The extent of gingivitis and periodontitis was assessed by taking measurements at the gingival margin using a periodontal probe. A gingivitis score between 0 and 4 was recorded for every tooth using a modified combination of the gingival index and sulcus bleeding index [29] (Table 4). Probing depth, gingival recession and furcation exposure were recorded according to the criteria in Table 5. Probing depth was measured from the gingival margin to the bottom of the periodontal pocket. Gingival recession was measured from the cementoenamel junction to the gingival margin.

Both were measured using the graduations of a periodontal probe. Total attachment loss was calculated as the sum of the gingival recession and the periodontal probing depth in accordance with established protocols [31, 32]. PD2 was classified as being up to 25% attachment loss and periodontitis stage 3 (PD3) as between 25 and 50% attachment loss, as defined by the American Veterinary Dental College. A dental chart was completed for each dog where, in addition to recording clinical status of each individual tooth as described above, missing teeth, crown fractures below the gingival margin and foreign bodies were documented. Dental radiographs were taken of some of the dogs and were inspected by a Diplomate of the EVDC to confirm the measures used in this study were indicative of early bone loss.

**Table 4:** Summary of metadata for each of the four locations

	UK	USA	China	Thailand
**Age (years)**				
Average	5.78	5.61	5.02	2.67
(95% CI)	(5.26, 6.3)^b^	(4.93, 6.29)^b^	(4.17, 5.87)^b^	(2.16, 3.18)^a^
Range	1.5 to 15	1 to 14.5	1 to 14	0.8 to 14
**Breed size**				
Small (< 10 kg)	36 (17.6%)	56 (46.3%)	57 (42.5%)	22 (17.7%)
Medium (10–25 kg)	76 (36.9%)	24 (19.8%)	43 (32.1%)	81 (65.3%)
Large (> 25 kg)	93 (45.1%)	41 (33.9%)	33 (24.8%)	21 (16.9%)
**Sex**				
Female	94 (45.2%)	54 (44.6%)	55 (45.1%)	59 (47.6%)
Male	114 (54.5%)	67 (54.5%)	67 (54.5%)	65 (52.4%)

^a, b^Significance with Tukey HSD homogenous groups at 5% within model

**Table 5:** Periodontitis scoring criteria

AVDC Stage^a^	Periodontal Probing Depth	Gingival Recession	Furcation Exposure
Stage 2 (PD2) Early periodontitis (< 25% attachment loss)	Large dog ≥3 (≥6 on canine teeth)	> 0	Grade 1; feel an indentation between the roots and the probe may advance 1 mm.
	Medium dog ≥3 (≥3 on canine teeth)		
	Small dog ≥2 (≥3 on canine teeth)		
Stage 3 (PD3) Moderate periodontitis (25–50% attachment loss)	Large dog > 5 (> 9 on canine teeth)	Large dog > 5 mm	Grade 2; obvious indentation between the roots and probe advances 50%.
	Medium dog > 4 (> 5 on canine teeth)	Medium & small dog > 3 mm	
	Small dog > 4 (> 4 on canine teeth)		

Large, medium and small dogs were defined as > 25 kg, 10-25 kg and < 10 kg respectively

^a^https://avdc.org/avdc-nomenclature/

### Sample collection

Subgingival plaque samples were collected at the time of clinical assessment. A sterile periodontal probe was gently inserted under the gingival margin and swept along the base of the crown. For samples collected in the USA, China and Thailand plaque was collected from all the teeth in the mouth and placed into a single Eppendorf tube containing 300 μl TE buffer (10 mM Tris-buffer, 1 mM EDTA, pH 8). If the tooth being sampled was PD3 or there was something that was of concern, for example a foreign body or an oral mass, then the sample was not collected from that tooth. In this instance, to prevent potential contamination of the sample, the probe was changed prior to collecting from other teeth and the exceptions recorded.

To limit the potential bias based on whole-mouth periodontal status, the samples were classified as health, gingivitis or periodontitis. Health was defined as less than two indicator teeth, or less than six teeth in total, with gingivitis. Gingivitis was defined as ≥2 indicator teeth, or six teeth in total, with gingivitis. Early periodontitis was defined as ≥3 indicator teeth, or ≥ 6 teeth in total, classified as PD2.

The samples were frozen at -20 °C within 10 min of collection. In the USA only, samples were stored for a maximum of 6 h at -20 °C and then transported to a central -80 °C facility. This type of facility was not available at the other three collections sites and therefore samples were stored at -20 °C until transported to the laboratory on dry ice for microbial analysis.

For the UK, samples were collected slightly differently [9]. For a mouth scored as health or gingivitis, 18 teeth in total were sampled (upper 03–08, lower 04, 08, 09). For a mouth scored as PD2, 6 to 12 teeth in total were sampled (upper 03, 04, 08, lower 04, 08, 09). For seven of the UK samples the incisors were not sampled and in some instances, if teeth were missing or there was a fracture below the gingival margin, alternative teeth were sampled to ensure sufficient sample quantity; these exceptions were recorded. Fourteen of the samples from the original cross-sectional study were excluded from this study due to the fact that the dental charts for the dogs could not be located and therefore the health status of each individual tooth was not known. A summary of the samples utilised in this study is provided in Table 6.

**Table 6:** Subgingival plaque samples

Location	Number of samples	Teeth sampled	Storage conditions
UK	209	Subset of teeth	-20 °C
USA	121	Whole mouth	-20 °C then -80 °C
China	133	Whole mouth	-20 °C
Thailand	124	Whole mouth	-20 °C

### Microbiologic analysis

DNA was extracted from the plaque samples and the 16S rRNA gene amplified according to the method described by Davis et al. [9]. PCR reactions were purified, quantified and multiplexed 454-pyrosequencing libraries created by pooling PCR amplicons in equimolar amounts. Sequences were generated using the GS FLX Titanium series 454 DNA pyrosequencer (454 Life Sciences). All pre-preparation and sequencing was performed by Eurofins Genomics (Ebersberg, Germany). Uni-directional sequencing was initiated from adapter B on the reverse primers. A sequencing depth of 15,000 sequences per sample was targeted which was comparable to that used for the UK cross-sectional study [9].

### Sequence processing

The standard flowgram files (SFF) were initially filtered by selecting reads with at least 360 flows and truncating long reads to 720 flows. Reads were filtered and denoised using the AmpliconNoise software (version V1.21 [33, 34]). For the initial filtering step, reads were truncated when flow signals dropped below 0.7, indicative of poor quality. Subsequently, reads were denoised in three stages; 1) Pyronoise to remove noise from flowgrams resulting from 454 sequencing errors (PyronoiseM parameters -s 60, −c 0.01), 2) Seqnoise to remove errors resulting from PCR amplification (SeqNoiseM parameters –s 25, −c 0.08), 3) Perseus to detect and remove chimeras resulting from PCR recombination. The denoised sequences were then clustered using QIIME v1.7.0. The QIIME script pick_otus.py, which utilises the Uclust v1.2.22q software program, was used to cluster sequences with ≥98% identity [35]. Uclust was run with modified parameters, with gap opening penalty set to 2.0 and gap extension penalty set to 1.0 and –A flag to ensure optimum alignment [35]. Representative sequences of all observed OTUs were annotated using BLAST [36] against the Silva SSU database release 119 [37]. If the alignment matched the top BLAST hit with ≥98% sequence identity and ≥ 98% sequence coverage then a species level was assigned but if these criteria were not met the next appropriate level of taxonomic assignment was allocated: ≥94% genus; ≥92% family; ≥90% order, ≥85% class, ≥80% phyla.

#### Phylogenetic tree construction

OTU representative sequences were blasted against Silva SSU database release 119, best hit full length 16S sequences were used to represent OTUs within the tree. An initial multiple sequence alignment (msa) was constructed using pynast v0.1 [38] and Greengenes core template, this alignment was filtered using filter_alignment.py script from QIIME v1.8.0 [35] with default settings. The filtered msa was realigned using MUSCLE v3.8.31 [39] with default settings and a phylogenetic tree inferred using FastTree v2.1.7 [40] using a generalised time-reversible model. The tree figure was plotted using GraPhlAn v0.9.7 (unpublished).

### Statistical analysis

An OTU was classed as rare if none of the geographical locations had an average proportion above 0.05% and/or had presence in less than two samples. The 0.05% cut-off was selected based on statistical analysis of data from mock communities [9].

#### Baseline analysis

Total sequence depth, the age (years) and average gingivitis score were analysed by generalised least squares linear models with a fixed effect of geographical location and weighting the variance by geographical location. Means were compared using Tukey HSD tests to the 5% level and reported with 95% family-wise confidence intervals. The percentage of rare sequences, healthy teeth and periodontitis teeth were analysed by logistic regression analyses (GLM with a quasi-binomial distribution and logit link) for proportions, using the count of rare sequences out of the total sequence depth. Geographical location was investigated as a fixed effect and means were compared using Tukey HSD tests to the 5% level. Contingency tables of breed size, sex and neuter status by geographical location were analysed using Chi-square tests for independence using a test level of 5%.

#### Univariate analysis

The 280 individual OTUs were analysed univariately by logistic regression analyses (GLM with a quasi-binomial distribution and logit link) for proportions, using the count for the OTU out of the total number of sequences. To enable model convergence when an OTU has many zero counts, 2 counts were added to each OTU count and 4 counts were added to the total count (analogous to adding 2 successes and 2 failures [41]) prior to analyses. The models were explored to investigate the correlation of the OTUs with the fixed effects for oral health status, as measured by the percentage of healthy teeth, percentage of periodontitis teeth and the average gingivitis score, and their two way interactions with geographical location and each other. Age and breed size were also explored as covariates. Models were built initially to minimise the quasi - Akaike’s Information Criterion (qAIC) for binomial distributions with over dispersion [42], with geographical location fixed to remain in the model. The model with the smallest qAIC were then chosen to be tested for significance. To adjust for multiplicity effect, the *p*-values of each fixed effect in the minimum qAIC model were adjusted according to the false discovery method of Benjamini and Hochberg [30] for the 280 OTUs analysed. Subsequently effects were removed from the model if found to be non-significant by Benjamini and Hochberg with a false discovery rate of 5% level. Once this minimal model by qAIC and Benjamini and Hochberg adjustment was formed, the data were subjected to a permutation test to assess the sensitivity of the results to possible outliers or deviations from the assumption of the generalised linear model. An effect remained in the model if the proportion of permutations where the significance of the effect was as least as small as the observed effect was less than 5%. Means and odds ratios between levels were then calculated at the covariate averages according to final model found, with 95% family wise confidence intervals. *P*-values for comparisons between geographical locations and breed sizes are calculated using a family-wise error rate of 5%.

#### Shannon diversity

The Shannon Diversity index of each sample was calculated [43] and analysed by linear regression modelling. Models were built using stepwise regression to minimise the AIC, with fixed effects as defined for the univariate analyses and the total number of sequences and geographical location fixed in the models. The data were then subjected to a permutation test according to the minimised model, as for univariate analyses. A test level of 5% was used.

#### Multivariate analysis

The log_10_ (proportions) (after 2 counts were added to each OTU count and 4 counts were added to the total count) for the profile were analysed by PCA. Score plots of the components were investigated for correlations with breed size, age, gender, neuter status, average gingivitis score and the percentage of healthy and periodontitis teeth in the mouth and in the sampled teeth.

Statistical analyses were performed in R v3.2.2 statistical software [44]. PCA analysis was performed using the library *vegan*, comparisons and confidence intervals were calculated using *multcomp* and the model AICs were calculated using *MuMIn*. Graphics were generated using *ggplot2* [45].

## Supplementary Information

**Additional file 1: Table S1.** OTUs that had significant interactions with location and other covariates. A) Age by location, B) average gingivitis by location, C) breed size by location, D) age by average gingivitis, E) age by percentage healthy teeth, F) age by percentage periodontitis teeth, G) average gingivitis by breed size, H) average gingivitis by percentage healthy teeth, I) average gingivitis by percentage periodontitis teeth, J) breed size by percentage healthy teeth, K) breed size by percentage healthy teeth, L) Percentage healthy teeth by percentage periodontitis teeth and M) age by breed size. Tables summarise estimated mean proportions, odds ratios, 95% confidence intervals and *p*-values.

**Additional file 2: Table S2.** Table summarises estimated mean proportions, odds ratios, 95% confidence intervals and *p*-values for OTUs that had significant main effects of A) location, B) age, C) average gingivitis, D) breed size, E) Percentage healthy teeth and F) percentage periodontitis teeth.

**Additional file 3: Figure S1.** A) proportion of healthy teeth, B) average gingivitis score and C) proportion of periodontitis teeth in sampled teeth compared to whole mouth coloured by location.

**Additional file 4:** **Figure S2.** Heatmap of OTU relative abundance. From left to right; hierarchical cluster of OTU presence/absence using Jaccard distance and average agglomeration, clusters of interest are coloured red (cluster A), blue (cluster B) and green (cluster C). OTU best hit annotations and phylum level classification denoted by coloured blocks. The percentage of all samples containing each OTU (presence defined as > 0.05%) is displayed using black to white shaded ribbon with a continuous scale where black would be an OTU present in all samples. The heatmap graphic represents the relative abundance of each OTU in each sample, ordered by sample country of origin, black represents relative abundances greater than 5% and white 0%.

## Data Availability

Data supporting the conclusions of this manuscript are available from the corresponding author upon reasonable request.

## Abbreviations

OTU: Operational taxonomic unit
PD2: Periodontal disease stage 2
PD3: Periodontal disease stage 3
UK: United Kingdom
USA: United States of America
V1: Variable region 1
V3: Variable region 3
GLM: Generalised linear model
Sp: Species
COT: Canine Oral Taxon
PCR: Polymerase chain reaction
DNA: Deoxyribonucleic acid
qAIC: Akaike’s Information Criterion
PCA: Principal components analysis
