# Supplementary figures and images for: Subgingival microbiota of dogs with healthy gingiva or early periodontal disease from different geographical locations
Source: BMC Vet Res. 2021 Jan 6;17:7. doi: 10.1186/s12917-020-02660-5 (PMC7789547; doi:10.1186/s12917-020-02660-5)

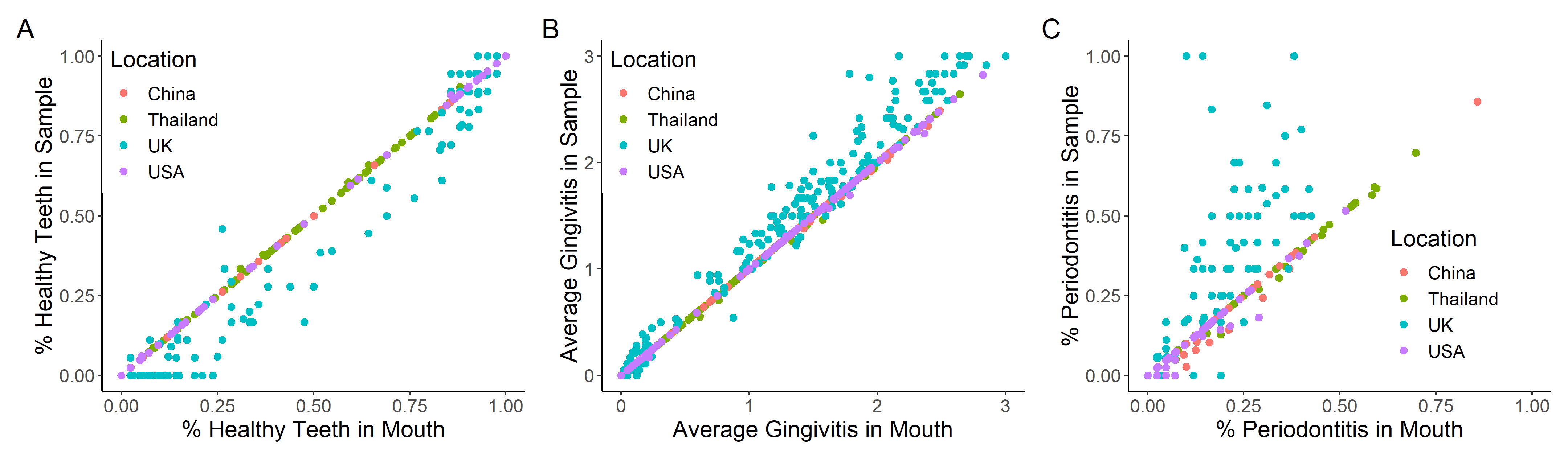

Supplement: Supplementary file 3 — Additional file 3: Figure S1. A) proportion of healthy teeth, B) average gingivitis score and C) proportion of periodontitis teeth in sampled teeth compared to whole mouth coloured by location. [file 12917_2020_2660_MOESM3_ESM.png]
