# Supplementary material for: Subgingival microbiota of dogs with healthy gingiva or early periodontal disease from different geographical locations
Source: BMC Vet Res. 2021 Jan 6;17:7. doi: 10.1186/s12917-020-02660-5 (PMC7789547; doi:10.1186/s12917-020-02660-5)
Supplement: Supplementary file 4 — Additional file 4: Figure S2. Heatmap of OTU relative abundance. From left to right; hierarchical cluster of OTU presence/absence using Jaccard distance and average agglomeration, clusters of interest are coloured red (cluster A), blue (cluster B) and green (cluster C). OTU best hit annotations and phylum level classification denoted by coloured blocks. The percentage of all samples containing each OTU (presence defined as > 0.05%) is displayed using black to white shaded ribbon with a continuous scale where black would be an OTU present in all samples. The heatmap graphic represents the relative abundance of each OTU in each sample, ordered by sample country of origin, black represents relative abundances greater than 5% and white 0%. [file 12917_2020_2660_MOESM4_ESM.pdf]

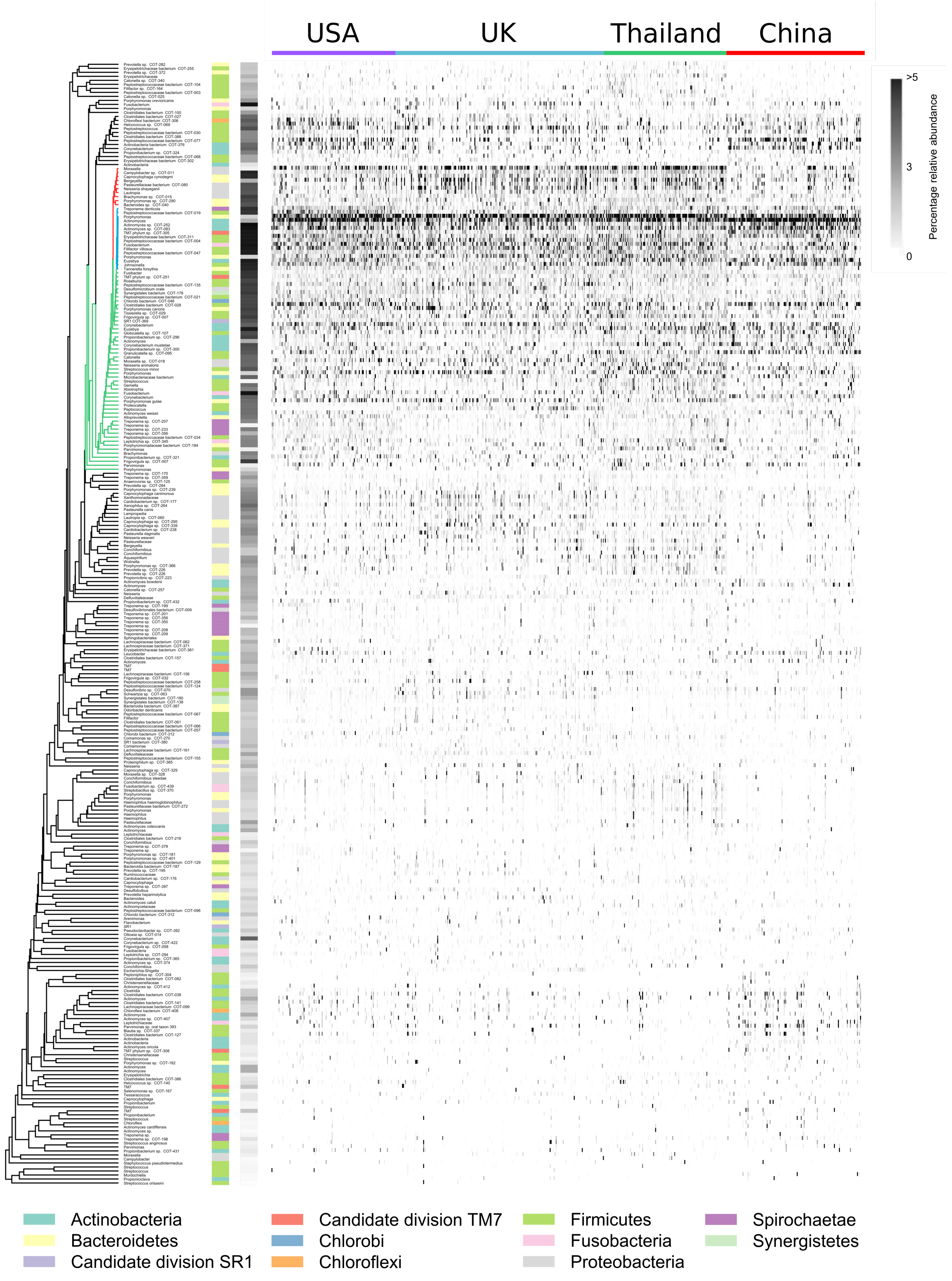
USA
UK
Thailand
China
>5
3
0
Percentage relative abundance
Actinobacteria
Bacteroidetes
Candidate division SR1
Candidate division TM7
Chlorobi
Chloroflexi
Firmicutes
Fusobacteria
Proteobacteria
Spirochaetae
Synergistetes
